# The association between atherogenic index of plasma and postoperative delirium in cardiac surgery patients: an analysis of the MIMIC-IV database

**DOI:** 10.1186/s13741-026-00717-x

**Published:** 2026-07-08

**Authors:** Xiaqing Zhang, Mingliang Xing, Afen Zhang, Rongzhi Zheng, Huiru Hu, Xianming Zeng

**Affiliations:** 1 First Department of Anesthesiology, Affiliated Hospital of Shaanxi University of Traditional Chinese Medicine, No.2 Weiyang West Road, Xianyang City, Shaanxi Province 712000 People’s Republic of China; 2 Department of Thoracic Surgery, The 2nd Affiliated Hospital of Air Force Medical University of PLA, No. 1 Xinsi Road, Baqiao District, Xi’an City, Shaanxi Province 710038 People’s Republic of China; 3 Department of Intensive Care Unit, Affiliated Hospital of Subei Health Management Center, Zhongshan Hospital, Fudan University, No. 9, Xinqu Avenue, Dafeng District, Yancheng City, Jiangsu Province 224100 People’s Republic of China

**Keywords:** Postoperative Delirium, Cardiac Surgery, Atherogenic Index of Plasma, MIMIC-IV

## Abstract

**Background:**

This study aimed to investigate the relationship between the atherogenic index of plasma (AIP) and the risk of postoperative delirium (POD) in patients undergoing cardiac surgery.

**Methods:**

A retrospective cohort study was conducted using data from the MIMIC-IV 2.2 with patients who underwent cardiac surgery. Propensity score matching (PSM) was used in a 1:1 ratio to balance baseline characteristics (age, sex, race) between delirium and non-delirium groups. Multivariable logistic regression models were employed to assess the independent association between AIP (analyzed as both a continuous and categorical variable in quartiles) and POD, with adjustments for demographics, comorbidities, laboratory parameters, vital signs, medication use, and surgical details. Restricted cubic spline (RCS) were used to explore nonlinearity. Subgroup and mediation analyses were also performed.

**Results:**

Among 6,067 eligible patients, 1,565 (25.8%) developed POD. After PSM, 3,130 patients were analyzed. Multivariable regression revealed a significant positive association between AIP and POD (OR: 1.622, 95% CI: 1.455–1.81, *P* < 0.001). Quartile stratification revealed that higher AIP levels were associated with an increased risk of POD (OR: 1.708, 95% CI: 1.399–2.088, *P* < 0.001). In the fully adjusted model (Model 3), AIP remained significantly associated with POD (OR: 1.435, 95% CI: 1.249–1.652, *P* < 0.001). RCS analysis revealed a significant nonlinear relationship between AIP and POD (*P* < 0.001), consistent across gender subgroups. Subgroup analysis revealed no significant interactions. Mediation analysis indicated that hemoglobin (Hb), red cell distribution width (RDW), albumin (Alb), and respiratory rate (RR) partially mediated the association between AIP and POD.

**Conclusion:**

AIP was independently and nonlinearly associated with an increased risk of POD following cardiac surgery. As an easily calculable composite lipid indicator, AIP may serve as a potential biomarker for preoperative risk stratification.

**Supplementary Information:**

The online version contains supplementary material available at https://doi.org/10.1186/s13741-026-00717-x.

Postoperative delirium (POD) is a frequent complication after surgery, particularly in patients aged 65 and older, with an incidence rate as high as 20%−50% following major surgeries such as cardiac procedures (Brown et al. 2016). POD is not only linked to extended hospital stays, higher healthcare costs, but also serves as an independent risk factor for long-term cognitive decline and increased postoperative mortality (Yan et al. 2023; Evered et al. 2018). Although its pathophysiological mechanisms involve multiple pathways such as neuroinflammation, oxidative stress, and neurotransmitter imbalances, effective prevention and treatment options remain limited. Consequently, the current focus of POD management has shifted toward preoperative risk prediction and stratification, aiming to improve postoperative outcomes by identifying high-risk patients early and implementing targeted interventions (Jin et al. 2020).

Within this context, identifying convenient and reliable preoperative predictive biomarkers has become a research hotspot. Existing investigations have identified several candidates, primarily systemic inflammatory indicators (e.g., interleukin-6, C-reactive protein) and neuroaxonal injury markers (e.g., neurofilament light chain, glial fibrillary acidic protein) (Mosharaf et al. 2025; Su et al. 2023). Despite their predictive potential for POD, the clinical utility of these biomarkers is restricted. Neuroinjury markers involve high costs and are not routinely performed, while acute inflammatory markers are easily influenced by surgical stress and preoperative fluctuations, failing to stably represent baseline metabolic vulnerability (Shan et al. 2025). Thus, a predictive tool leveraging routine biochemical parameters to reflect chronic pathological states is urgently required in clinical practice.

The relationship between lipid dysregulations and POD has gradually attracted the attention of researchers in recent years. The brain is one of the body's most lipid-rich organs, with lipids playing a critical role in maintaining neuronal membrane integrity, synaptic function, and signal transduction (Vitali et al. 2014). Both preclinical and clinical studies have revealed that POD patients often exhibit specific lipid profile dysregulation, such as alterations in levels of omega-3 polyunsaturated fatty acids, cholesterol, and sphingolipids (Huang et al. 2022; Qian et al. 2020). These lipid dysregulations may increase the brain's vulnerability to perioperative stress by exacerbating neuroinflammation, compromising blood–brain barrier integrity, and disrupting energy metabolism, ultimately triggering delirium (Shaw et al. 2025).

The atherogenic index of plasma (AIP), is mathematically defined as the logarithmic ratio of triglycerides to high-density lipoprotein cholesterol, it not only reflects levels of triglycerides (TG) and high-density lipoprotein cholesterol (HDL-C), but also serves as a powerful predictor of dyslipidemia (Dobiásová 2001; Nam et al. 2020). Notably, recent longitudinal evidence has linked higher AIP levels to an increased risk of cognitive impairment in middle-aged and elderly populations, suggesting its potential as a stable biomarker for neurocognitive risk (Zhou et al. 2025).

Nevertheless, the relationship between AIP and POD remains unclear. Given the significant overlap between the dyslipidemia represented by AIP and the potential pathophysiological mechanisms of POD, we hypothesize that elevated preoperative AIP independently increases the risk of POD in cardiac surgery patients. To validate this hypothesis, this study utilizes the large public database MIMIC-IV to systematically investigate the association between preoperative AIP and POD in cardiac surgery, aiming to provide a novel, readily accessible laboratory marker for perioperative risk stratification.

## Methods

### Data source

This research utilized a retrospective cohort design, sourcing data from the Medical Information from Critical Care (MIMIC-IV) database (version 2.2) to obtain research data (Johnson et al. 2023). MIMIC-IV is a publicly accessible and one of the most comprehensive intensive care databases in critical care medicine. The dataset comprises comprehensive clinical data for patients hospitalized at Beth Israel Deaconess Medical Center (BIDMC) between 2008 and 2019, encompassing length of stay, laboratory test outcomes, medication regimens, vital signs, and other pertinent clinical information. The Institutional Review Boards of the Massachusetts Institute of Technology and Beth Israel Deaconess Medical Center (Boston, Massachusetts, USA) granted permission to use the MIMIC-IV database for this research.

### Ethics and data privacy

This research data has been de-identified to uphold ethical standards and protect patient privacy, with all necessary measures implemented to ensure confidentiality. The Institutional Review Board at Beth Israel Deaconess Medical Center waived the informed consent requirement due to the data being de-identified. Author Xiaqing Zhang successfully completed the National Institutes of Health (NIH) online course “Protection of Human Research Participants” (ID: 15008001) and was therefore authorized to access the MIMIC-IV database for data extraction.

### Data inclusion and exclusion criteria

**Fig. 1:**
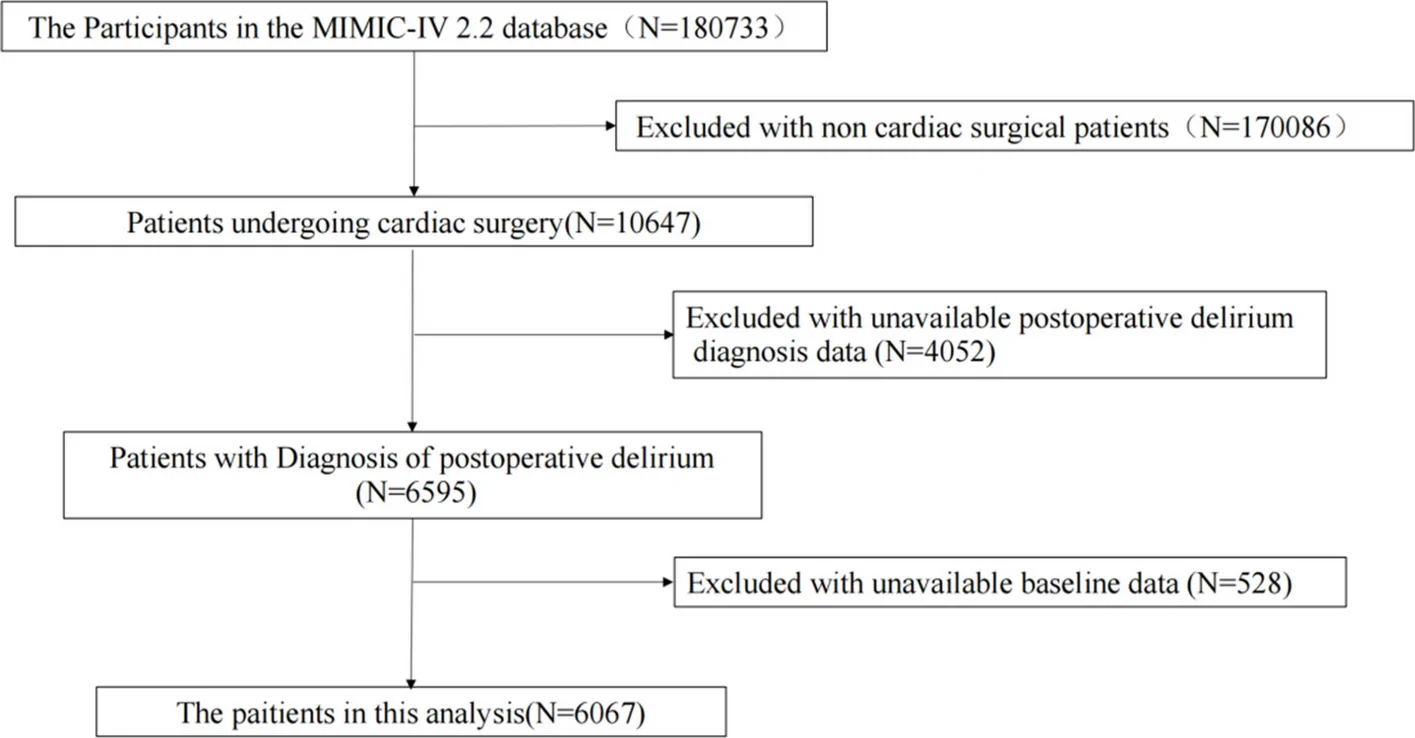
Flowchart of patient selection

The MIMIC-IV database contains clinical data from 180,733 patients admitted to the hospital's intensive care unit (ICU) between 2008 and 2019. This study involved patients identified through International Classification of Diseases, Ninth or Tenth Revision (ICD-9/10) codes as having undergone cardiac surgery or experienced delirium (Supplementary Table S1). From the initial cohort of 180,733 patients, we excluded cases for the following reasons: (1) removal of non-cardiac surgery diagnoses (*N* = 170,086), (2) removal of patients with unavailable postoperative delirium diagnosis data (*N* = 4,052), and (3) removal of cases lacking baseline data (*N* = 528). The final study cohort comprised 6,067 patients, including 1,565 delirium patients and 4,502 non-delirium patients (Fig. 1).

### Data extraction

Clinical data were retrieved from the MIMIC-IV database using structured query language (SQL). The extracted data encompassed demographic details, vital signs, essential laboratory parameters, and significant comorbidities.

Specifically, demographic details included age, gender, and ethnicity. Vital signs comprise systolic blood pressure (SBP), diastolic blood pressure (DBP), heart rate (HR), respiratory rate (RR), and pulse oximetry oxygen saturation (SpO_2_). Laboratory parameters primarily include hematologic indicators, hepatic and renal function markers, and electrolyte levels. Additionally, we documented significant comorbidities potentially linked to cardiac surgery and POD, include cerebrovascular disease, neurological disease, respiratory system disease, diabetes, hypertension, congestive heart failure, myocardial infarction, kindey disease, liver disease, obstructive sleep apnea (Devinney et al. 2022; Aldecoa et al. 2017; Li et al. 2025; Dilmen et al. 2024). And we also extracted data on the type of cardiac surgery and the use of anesthetic sedatives potentially related to POD occurrence. For all variables, we utilized the first recorded value after patient admission.

### Definition of AIP

AIP is determined by assessing plasma levels of HDL-C and TG (Dobiásová 2001). The AIP is calculated using the formula: log₁₀ [TG (mmol/L) / HDL-C (mmol/L)].

### Statistical analysis

Data processing and analysis were conducted using R software (version 4.1.1). Continuous variables with non-normal distribution are described using medians and interquartile ranges (IQR) for baseline characteristic analyses. Categorical variables are reported as counts and weighted percentages. The Wilcoxon rank-sum test assessed differences in continuous variables across AIP quartiles, while the Rao-Scott chi-square test evaluated weighted percentages of categorical variables.

Propensity score matching (PSM) was conducted in a 1:1 ratio based on sex, age, and race to ensure comparable baseline data distribution between the delirium and non-delirium groups, minimizing baseline data impact on research outcomes. Odds ratios (OR) and 95% confidence intervals (CI) were derived using logistic regression models. Covariate selection was guided by established associations with POD, clinically significant baseline differences between groups (*P* < 0.01), and clinical relevance. AIP was incorporated into the regression models both as a continuous variable and as a categorical variable. As a continuous variable, the raw AIP value was entered into the model. The original AIP values were divided into quartiles based on the interquartile range, ranked from low to high. These quartiles were labeled as the first quartile (Q1), second quartile (Q2), third quartile (Q3), and fourth quartile (Q4), and were further evaluated as categorical variables. When AIP was treated as a categorical variable, Q1 was used as the reference group. Three distinct statistical models were constructed: Model 1 represented the unadjusted model. Model 2 was adjusted for the following factors: ICU length of stay, in-hospital mortality, comorbidities, sedative drug use, and surgery category. Model 3 was further adjusted based on Model 2 by incorporating laboratory biochemical parameters and vital signs. Survey weights were applied to all regressions, and continuous covariates with non-normal distributions were transformed using weighted quartiles.

Restricted cubic spline (RCS) analysis was employed to explore potential non-linear relationships between AIP and the risk of POD. Subgroup analysis was conducted to assess whether the association between AIP and POD after cardiac surgery differed across various subgroups, with results visualized using forest plots. Interaction analysis was performed to evaluate potential interactions between each subgroup and AIP. *P*-values were corrected for the False Discovery Rate (FDR). A two-sided *P*-value < 0.05 was considered statistically significant.

## Results

### Baseline characteristics of patients

From an analysis of 180,733 patients in the MIMIC-IV database, 6,067 patients met the study's criteria and were included. Figure 1 shows the flow diagram of the patient selection process. with 1,565 patients (25.8%) experiencing POD during hospitalization. After PSM, the analysis included 3,130 patients, evenly divided into two groups of 1,565 each. Post-matching showed there were no significant differences between the two groups in terms of demographic characteristics, including age, sex, and race (*P* > 0.05). However, significant differences persisted in comorbidities (cerebrovascular disease, neurological disease, respiratory system disease, diabetes, congestive heart failure, myocardial infarction, kidney disease, and obstructive sleep apnea), surgical category, sedative use (dexmedetomidine and propofol), laboratory parameters, vital signs, ICU length of stay, in-hospital mortality, and AIP (all *P* < 0.05). Notably, the AIP remained significantly higher in the delirium group compared with the non-delirium group after matching (1.05 [IQR, 0.73] vs. 0.86 [IQR, 0.58]; *P* < 0.001). Baseline characteristics are presented in Table 1.

**Table 1:** Baseline characteristics of patients

Variable	Before PSM			*p*-value^2^	After PSM			*p*-value^2^
	Overall	Non-Delirium	Delirium		Overall	Non-Delirium	Delirium	
	*N* = 6,067^1^	*N* = 4,502^1^	*N* = 1,565^1^		*N* = 3,130^1^	*N* = 1,565^1^	*N* = 1,565^1^	
Age	69 (10)	69 (10)	69 (10)	0.13	69 (10)	68 (10)	69 (10)	0.1
Gender				0.034				> 0.99
Male	3,753(61.9%)	2,820 (63%)	933 (60%)		1,866(60%)	933 (60%)	933 (60%)	
Female	2,314(38.1%)	1,682 (37%)	632 (40%)		1,264(40%)	632 (40%)	632 (40%)	
Race				0.15				0.38
Asian	109 (1.8%)	87 (1.9%)	22 (1.4%)		52 (1.7%)	30 (1.9%)	22 (1.4%)	
Black	276 (4.5%)	194 (4.3%)	82 (5.2%)		150 (4.8%)	68 (4.3%)	82 (5.2%)	
White	4,586(75.6%)	3,422 (76%)	1,164(74%)		2,348(75%)	1,184 (76%)	1,164(74%)	
Others	1,096(18.1%)	799 (18%)	297 (19%)		580 (19%)	283 (18%)	297 (19%)	
Cerebrovascular disease	377 (6.2%)	156 (3.5%)	221 (14%)	< 0.001	275 (8.8%)	54 (3.5%)	221 (14%)	< 0.001
Neurological disease	130 (2.1%)	50 (1.1%)	80 (5.1%)	< 0.001	96 (3.1%)	16 (1.0%)	80 (5.1%)	< 0.001
Respiratory system disease	860 (14%)	535 (12%)	325 (21%)	< 0.001	514 (16%)	189 (12%)	325 (21%)	< 0.001
Diabetes	2,414 (40%)	1,688 (37%)	726 (46%)	< 0.001	1,345(43%)	619 (40%)	726 (46%)	< 0.001
Hypertension	2,370 (39%)	1,703 (38%)	667 (43%)	< 0.001	1,279(41%)	612 (39%)	667 (43%)	0.046
Congestive Heart Failure	1,143 (19%)	705 (16%)	438 (28%)	< 0.001	686 (22%)	248 (16%)	438 (28%)	< 0.001
Myocardial Infarction	1,856 (31%)	1,276 (28%)	580 (37%)	< 0.001	1,014(32%)	434 (28%)	580 (37%)	< 0.001
Kindey disease	269 (4.4%)	120 (2.7%)	149 (9.5%)	< 0.001	189 (6.0%)	40 (2.6%)	149 (9.5%)	< 0.001
Obstructive sleep apnea	977 (16%)	670 (15%)	307 (20%)	< 0.001	530 (17%)	223 (14%)	307 (20%)	< 0.001
Surgical category				< 0.001				< 0.001
Aortic replacement	390 (6.4%)	126 (2.8%)	264 (17%)		312(10%)	48 (3.1%)	264 (17%)	
Combined cardiac surgery	1,797 (30%)	1,407 (31%)	390 (25%)		873 (28%)	483 (31%)	390 (25%)	
GABA	939 (15%)	728 (16%)	211 (13%)		471 (15%)	260 (17%)	211 (13%)	
Valve surgery	2,941 (48%)	2,241 (50%)	700 (45%)		1,474(47%)	774 (49%)	700 (45%)	
Dexmedetomidine	576 (9.5%)	289 (6.4%)	287 (18%)	< 0.001	386 (12%)	99 (6.3%)	287 (18%)	< 0.001
Propofol	5,484 (90%)	4,210 (94%)	1,274(81%)	< 0.001	2,739(88%)	1,465 (94%)	1,274(81%)	< 0.001
Hb (g/L)	11.52 (2.09)	11.72 (2.05)	10.95(2.08)	< 0.001	11.27(2.08)	11.58 (2.02)	10.95(2.08)	< 0.001
RDW (%)	14.90 (2.10)	14.73 (1.96)	15.38(2.39)	< 0.001	15.06(2.21)	14.74 (1.97)	15.38(2.39)	< 0.001
Lymphocyte (× 10⁹/L)	1.81 (2.91)	1.82 (2.46)	1.80 (3.95)	< 0.001	1.80 (3.12)	1.80 (1.97)	1.80 (3.95)	< 0.001
Monocyte (× 10⁹/L)	0.71 (0.40)	0.70 (0.39)	0.74 (0.43)	0.001	0.72 (0.42)	0.70 (0.41)	0.74 (0.43)	0.001
Neutrophil (× 10⁹/L)	7.9 (5.1)	7.7 (4.9)	8.4 (5.6)	0.003	8.1 (5.3)	7.8 (4.9)	8.4 (5.6)	0.02
WBC (× 10⁹/L)	9.7 (4.3)	9.5 (4.2)	10.2 (4.8)	< 0.001	9.8 (4.5)	9.4 (4.1)	10.2 (4.8)	< 0.001
BUN (mmol/L)	26 (18)	24 (16)	30 (22)	< 0.001	27 (19)	24 (16)	30 (22)	< 0.001
UA (μmol/L)	6.45 (2.37)	6.38 (2.28)	6.65 (2.59)	0.006	6.49 (2.44)	6.34 (2.28)	6.65 (2.59)	0.006
Cr (μmol/L)	1.31 (1.07)	1.25 (0.99)	1.49 (1.27)	< 0.001	1.37 (1.17)	1.25 (1.04)	1.49 (1.27)	< 0.001
Cl⁻ (mmol/L)	101.6 (5.1)	101.8 (4.9)	100.8 (5.5)	< 0.001	101.3 (5.3)	101.8 (4.9)	100.8 (5.5)	< 0.001
Alb (g/L)	3.80 (0.60)	3.88 (0.55)	3.59 (0.69)	< 0.001	3.73 (0.64)	3.88 (0.54)	3.59 (0.69)	< 0.001
Glu (mmol/L)	124 (45)	122 (43)	132 (49)	< 0.001	128 (47)	123 (45)	132 (49)	< 0.001
HDL (mmol/L)	48 (15)	49 (15)	46 (15)	< 0.001	47 (15)	48 (15)	46 (15)	0.003
HR(t/min)	82 (15)	82 (15)	83 (16)	0.065	82 (16)	81 (15)	83 (16)	0.042
RR(t/min)	17.9 (6.0)	17.4 (5.8)	19.3 (6.3)	< 0.001	18.4 (6.2)	17.5 (6.0)	19.3 (6.3)	< 0.001
SpO_2_(%)	97.59 (2.94)	97.71 (2.82)	97.25(3.22)	< 0.001	97.49(3.03)	97.74 (2.81)	97.25(3.22)	< 0.001
ICU length of stay	7 (11)	4 (4)	16 (18)	< 0.001	10 (14)	4 (4)	16 (18)	< 0.001
In-hospital death	153 (2.5%)	90 (2.0%)	63 (4.0%)	< 0.001	98 (3.1%)	35 (2.2%)	63 (4.0%)	0.004
AIP	0.90 (0.63)	0.84 (0.58)	1.05 (0.73)	< 0.001	0.96 (0.67)	0.86 (0.58)	1.05 (0.73)	< 0.001

*Hb* Hemoglobin, *RDW* Red Cell Distribution Width, *WBC* White Blood Cell, *BUN* Blood Urea Nitrogen, *UA* Uric Acid, *Cr* Creatinine, *Cl⁻* Chloride, *Alb* Albumin, *HDL* High—Density Lipoprotein, *Glu* Glucose, *HR* Heart Rate, *RR* Respiratory Rate, *SpO*_*2*_ Pulse Oximetry Oxygen Saturation, *AIP* Atherogenic Index of Plasma

1.Mean (SD) or Frequency (%)

2.Pearson’s Chi-squared test; Wilcoxon rank sum test; Fisher’s exact test

### Association between POD and AIP in cardiac surgery patients

The association between AIP and POD in cardiac surgery patients was analyzed using a multiple linear regression model (Table 2). Model 1 (without covariates) showed a positive correlation between AIP and POD (OR: 1.622, 95% CI: 1.455—1.81, *P* < 0.001). Furthermore, AIP quartile stratification revealed a significant difference between the Q4 group (OR: 1.708, 95% CI: 1.399–2.088, *P* < 0.001) and the Q1 group, indicating that higher AIP levels are associated with a higher risk of POD. Model 2 (after adjusting for covariates including ICU length of stay, in-hospital mortality, comorbidities [cerebrovascular disease, neurological disease, respiratory system disease, congestive heart failure, diabetes, hypertension, renal disease, myocardial infarction, obstructive sleep apnea], dexmedetomidine, propofol, and surgery category) further analyzed the relationship, showing that AIP remained significantly associated with POD (OR: 1.431, 95% CI: 1.247–1.644, *P* < 0.001). The Q4 quartile group demonstrated a significantly higher risk of POD compared to the Q1 quartile group (OR: 1.396, 95% CI: 1.089–1.792, *P* = 0.0085). Model 3 (further adjusting for Hb, RDW, lymphocytes, monocytes, neutrophils, WBC, BUN, UA, Cr, Cl⁻, Alb, Glu, RR, SpO_2_, and other clinical variables based on Model 2) was also observed a significant positive correlation between AIP and POD (OR: 1.435, 95% CI: 1.249–1.652, *P* < 0.001). In Model 3, the Q4 group demonstrated a notably higher risk of POD compared to the Q1 group (OR: 1.38, 95% CI: 1.073–1.777, *P* = 0.0122). Trend *P*-values showed significant trends with *P* < 0.001 in Model 1, *P* = 0.0141 in Model 2, and *P* = 0.0156 in Model 3.

**Table 2:** The relationship between AIP and delirium after cardiac surgery

Characterisitic		Model 1			Model 2			Model 3		
		OR	95%CI	*P*-value	OR	95%CI	*P*-value	OR	95%CI	*P*-value
AIP continuous		1.622	(1.455,1.81)	< 0.001	1.431	(1.247,1.644)	< 0.001	1.435	(1.249,1.652)	< 0.001
AIP quantile										
Q1(low)(< 0.41)	781(24.95%)	Ref	Ref		Ref	Ref		Ref	Ref	
Q2( 0.41- < 0.88)	781(24.95%)	1.086	(0.89,1.325)	0.4167	1.06	(0.829,1.354)	0.6437	1.041	(0.812,1.335)	0.7518
Q3( 0.41- < 0.88)	785(25.08%)	1.191	(0.977,1.453)	0.0844	1.049	(0.817,1.346)	0.7077	1.06	(0.823,1.366)	0.6509
Q4(high)(≥ 1.4)	783(25.02%)	1.708	(1.399,2.088)	< 0.001	1.396	(1.089,1.792)	0.0085	1.38	(1.073,1.777)	0.0122
*P* for trend				< 0.001			0.0141			0.0156

The model 1 was the crude model

The model 2 was adjusted by ICU length of stay, In-hospital death, Cerebrovascular disease, Neurological disease, Respiratory system disease, Congestive Heart Failure, Diabetes, Hypertension, Kidney disease, Myocardial Infarction, Obstructive sleep apnea, Dexmedetomidine, Propofol, Surgical category

The model 3 was further adjusted by ICU length of stay, In-hospital death, Cerebrovascular disease, Neurological disease, Respiratory system disease, Congestive Heart Failure, Diabetes, Hypertension, Kidney disease, Myocardial Infarction, Obstructive sleep apnea, Dexmedetomidine, Propofol, Surgical category, Hemoglobin, Red Cell Distribution Width, Lymphocyte, Monocyte, Neutrophil, White Blood Cell, Blood Urea Nitrogen, Uric Acid, Creatinine, Chloride, Albumin, Glucose, Respiratory Rate, Pulse Oximetry Oxygen Saturation

*OR* odds ratio, *CI* confidence intervals

### RCS curve for the association between AIP and POD

**Fig. 2:**
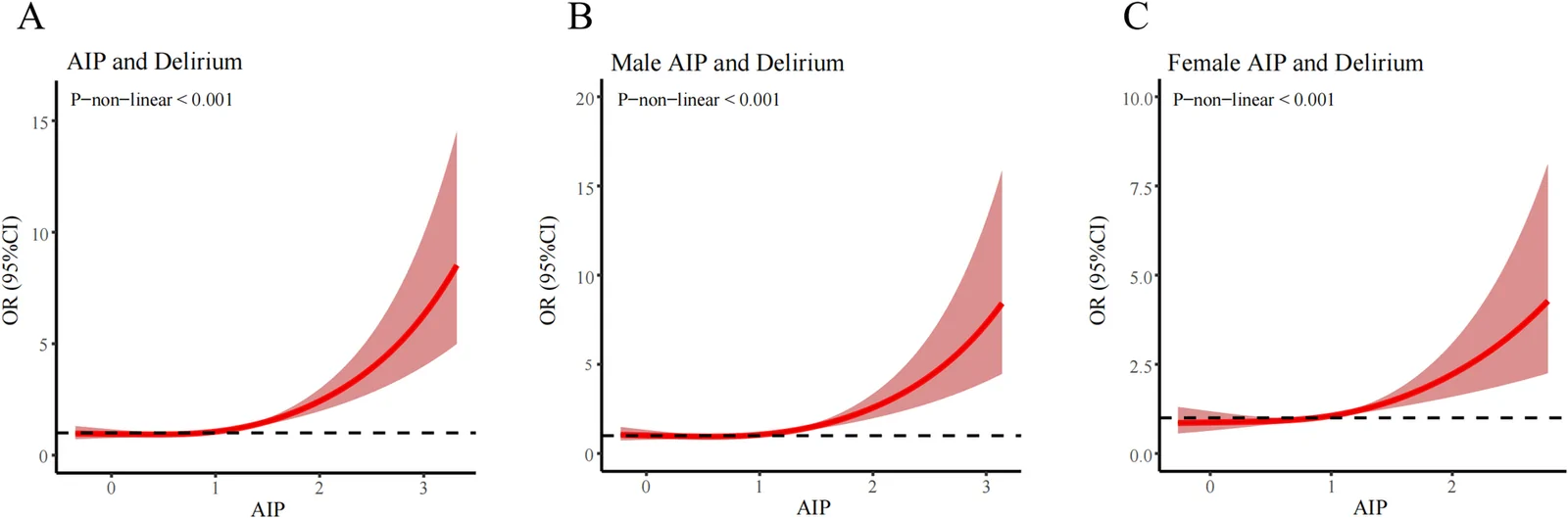
RCS curve for the association between AIP and POD. Note: the red bold line indicating the odds ratio and the shaded area representing the 95% CI

RCS curves were employed to examine the relationship between AIP and POD in cardiac surgery patients, adjusting for all relevant covariates (Fig. 2). The results revealed a notable nonlinear association between AIP and POD in cardiac surgery patients (*P* for non-linearity < 0.001, Fig. 2A). In gender subgroup analysis, a significant nonlinear relationship between AIP and POD observed in the male group (*P* for non-linearity < 0.001, Fig. 2B). In the female subgroup, AIP also showed a significant nonlinear relationship with POD (*P* for non-linearity = 0.015, Fig. 2C). These results further confirm the important role of AIP in the occurrence of POD in cardiac surgery patients and suggest that this non-linear relationship is robust and consistent across different genders.

### Relationship between AIP and baseline characteristics of subgroups

**Fig. 3:**
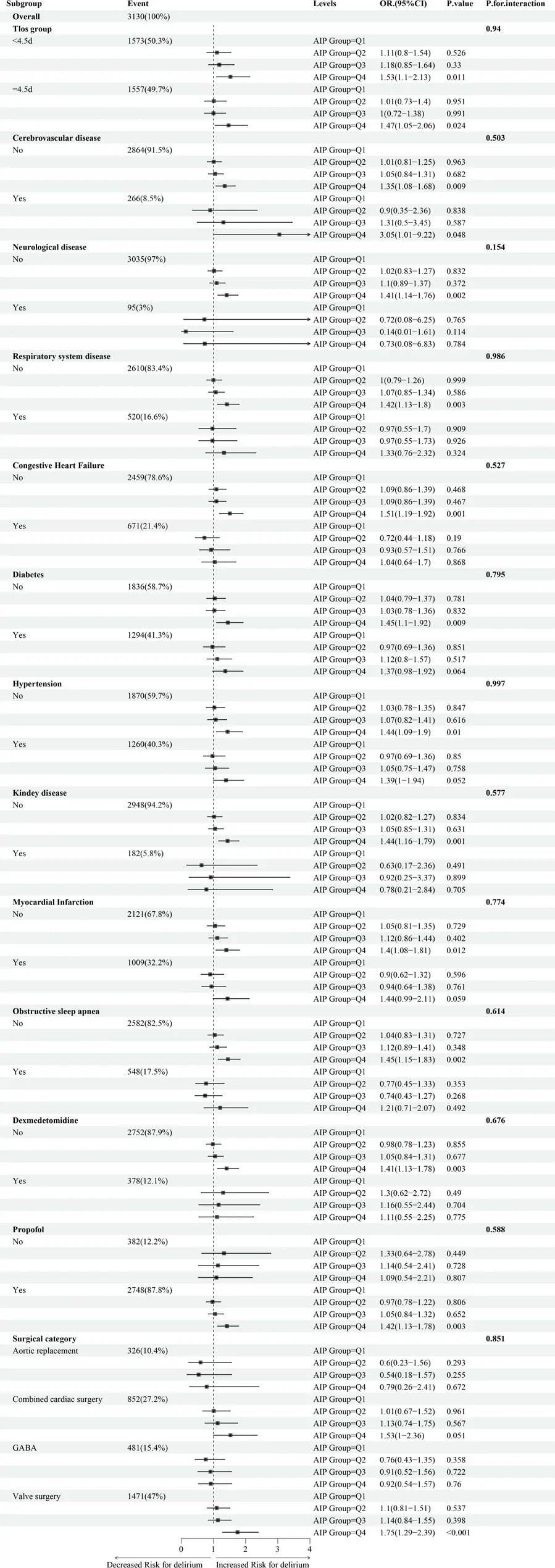
Forest plot of subgroup analysis. Note: Covariates: hemoglobin, red blood cell distribution width, lymphocyte count, monocyte count, neutrophil count, white blood cell count, blood urea nitrogen, uric acid, creatinine, chloride, albumin, glucose, respiratory rate, and pulse oximetry oxygen saturation

During subgroup analysis, multiple variables were evaluated, including ICU length of stay, cerebrovascular disease, neurological disorders, respiratory diseases, congestive heart failure, diabetes, hypertension, renal disease, obstructive sleep apnea, dexmedetomidine, propofol, and surgical category. The results indicated no significant interactions were observed among these subgroups (*P* for interaction > 0.05 for all) (Fig. 3). This further confirms that the association between AIP and POD remains consistent across subgroups.

### Association of relevant indicators with AIP and POD in cardiac surgery

Table 3 presents the association between AIP and relevant indicators, analyzed under three models—Model 1, Model 2, and Model 3—with a focus on indicators with *P* < 0.05. Among these, Alb showed a statistically significant association with AIP across all three models (*P* < 0.001 for all), with β values of −0.254, −0.179, and −0.177 respectively. and the progressively narrowing 95% CI indicate a strong negative correlation between AIP and Alb, with stable results. Cr exhibited *P* values of 0.001, 0.014, and 0.031 across the three models, with decreasing β values, suggesting a positive correlation between AIP and Cr that may weaken with model adjustments. BUN showed *P* < 0.05 in all three models (< 0.001, 0.007, 0.016), indicating a positive correlation. WBC, monocyte, Hb, RR, neutrophil, and RDW showed strong and statistically significant associations with AIP (*P* < 0.001 for all). A significant association was also found for Lymphocyte count in Model 1 (*P* = 0.021) and Model 2 (*P* = 0.047). Cl⁻ exhibited a *P* value of 0.043 only in Model 3, with no significant correlation in other models. UA and SpO_2_ exhibited *P* > 0.05 across all models, indicating no significant association between AIP and these variables.

**Table 3:** The associations between AIP and related indicators

Variable		β value	95% CI	*P*-value
Glu	Model1	0.001	(0,0.003)	0.058
	Model2	0.001	(−0.001,0.002)	0.22
	Model3	0.001	(−0.001,0.002)	0.257
Alb	Model1	−0.254	(−0.354,−0.154)	< 0.001
	Model2	−0.179	(−0.285,−0.074)	0.001
	Model3	−0.177	(−0.283,−0.071)	0.001
Cl⁻	Model1	0.007	(−0.005,0.019)	0.223
	Model2	0.012	(−0.001,0.024)	0.062
	Model3	0.012	(0,0.025)	0.043
Cr	Model1	0.099	(0.041,0.157)	0.001
	Model2	0.073	(0.015,0.132)	0.014
	Model3	0.065	(0.006,0.124)	0.031
UA	Model1	0.012	(−0.014,0.038)	0.358
	Model2	0.006	(−0.02,0.032)	0.64
	Model3	0.004	(−0.022,0.03)	0.752
BUN	Model1	0.007	(0.003,0.01)	< 0.001
	Model2	0.005	(0.001,0.008)	0.007
	Model3	0.004	(0.001,0.008)	0.016
WBC	Model1	0.039	(0.025,0.053)	< 0.001
	Model2	0.036	(0.022,0.051)	< 0.001
	Model3	0.036	(0.022,0.05)	< 0.001
Monocyte	Model1	0.354	(0.2,0.508)	< 0.001
	Model2	0.294	(0.139,0.45)	< 0.001
	Model3	0.294	(0.137,0.451)	< 0.001
Lymphocyte	Model1	0.022	(0.003,0.045)	0.021
	Model2	0.02	(0,0.044)	0.047
	Model3	0.019	(−0.001,0.043)	0.06
Hb	Model1	−0.065	(−0.095,−0.035)	< 0.001
	Model2	−0.046	(−0.077,−0.015)	0.004
	Model3	−0.048	(−0.079,−0.017)	0.002
SpO_2_	Model1	−0.011	(−0.032,0.009)	0.276
	Model2	−0.005	(−0.026,0.015)	0.608
	Model3	−0.004	(−0.025,0.016)	0.693
RR	Model1	0.018	(0.008,0.028)	< 0.001
	Model2	0.012	(0.001,0.023)	0.025
	Model3	0.012	(0.001,0.023)	0.025
Neutrophil	Model1	0.032	(0.02,0.045)	< 0.001
	Model2	0.028	(0.016,0.041)	< 0.001
	Model3	0.028	(0.016,0.041)	< 0.001
RDW	Model1	0.052	(0.024,0.08)	< 0.001
	Model2	0.035	(0.006,0.064)	0.017
	Model3	0.033	(0.004,0.062)	0.025

The model 1 was the crude model

The model 2 was adjusted by ICU length of stay, In-hospital death

The model 3 was adjusted by ICU length of stay, In-hospital death, Cerebrovascular disease, Neurological disease, Respiratory system disease, Congestive Heart Failure, Diabetes, Hypertension, Kidney disease, Myocardial Infarction, Obstructive sleep apnea, Dexmedetomidine, Propofol, Surgical category

*Glu* Glucose, *Alb* Albumin, *Cl⁻* Chloride, *Cr* Creatinine, *UA* Uric Acid, *BUN* Blood Urea Nitrogen, *WBC* White Blood Cell, *Hb* Hemoglobin, *SpO*_*2*_ Pulse Oximetry Oxygen Saturation, *RR* Respiratory Rate, *RDW* Red Cell Distribution Width

### Analysis of the mediating role in delirium after cardiac surgery

**Fig. 4:**
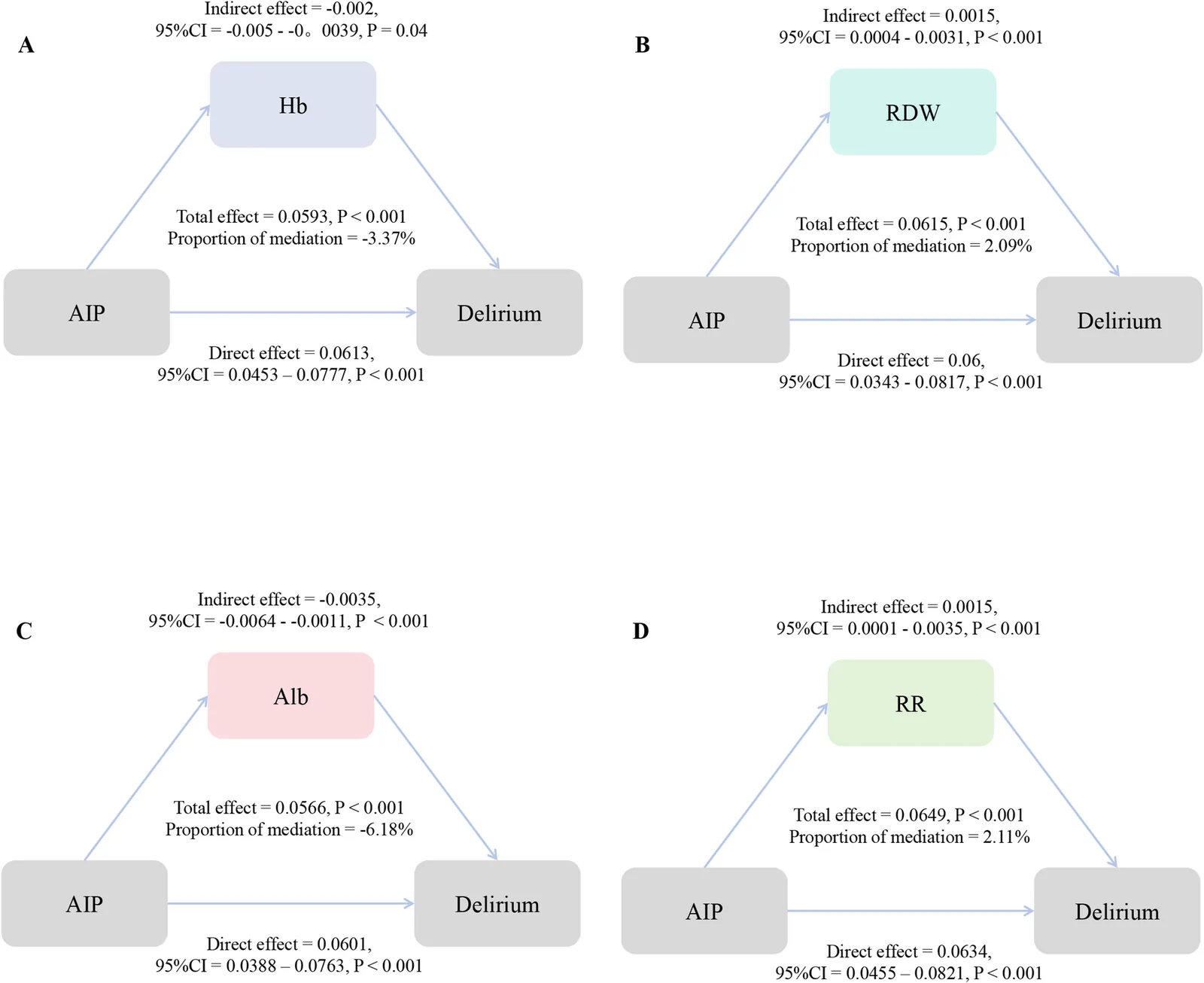
Analysis of the mediating role in delirium after cardiac surgery

We performed a mediation analysis to explore the influence of metabolic-related indicators on the relationship between AIP and POD after cardiac surgery, as indicated by our analysis of relevant indicators (Fig. 4). The findings revealed that with Hb as the mediating variable, the indirect effect of AIP on POD was −0.002 (95% CI: −0.005 to −0.00039, *P* = 0.04); The total effect was 0.0593 (*P* < 0.001), the mediation proportion was −3.37%, and the direct effect was 0.0613 (95% CI: 0.0453 to 0.0777, *P* < 0.001) (Fig. 4A). With RDW as the mediating variable, the indirect effect of AIP on POD was 0.0015 (95% CI: 0.0004 to 0.0031, *P* < 0.001). The total effect was 0.0615 (*P* < 0.001), the mediation proportion was 2.09%, and the direct effect was 0.06 (95% CI: 0.0343 to 0.0817, *P* < 0.001) (Fig. 4B). With Alb as the mediating variable, the indirect effect was −0.0035 (95% CI: −0.0064 to −0.0011, *P* < 0.001). The total effect was 0.0566 (*P* < 0.001), the mediation proportion was 6.18%, and the direct effect was 0.0601 (95% CI: 0.0388 to 0.0763, *P* < 0.001) (Fig. 4C). Finally, with RR as the mediator, the indirect effect of AIP on delirium was 0.0015 (95% CI: 0.0001 to 0.0035, *P* = 0.04). The total effect was 0.0649 (*P* < 0.001), the mediation proportion was 2.11%, and the direct effect was 0.0634 (95% CI: 0.0455 to 0.0821, *P* < 0.001) (Fig. 4D). See Supplementary Table S2 for details.

## Discussion

This research is the first exploration of the relationship between the AIP and POD. Results indicate that within a large-scale cardiac surgery cohort, that a preoperatively elevated AIP is independently associated with a higher risk of POD. This association remains robust after multivariate adjustment, and RCS analysis reveals a significant nonlinear “J-shaped” relationship between the two groups. Our findings suggest that preoperative dyslipidemia serves as a significant risk factor—likely reflecting a poorer overall health status—associated with increased postoperative brain vulnerability and susceptibility to delirium, providing a novel biomarker for identifying high-risk patients. AIP serves as an effective predictive tool for assessing preoperative POD risk, aiding clinical anesthesiologists in identifying high-risk patients and guiding perioperative risk stratification.

Lipids are widely distributed throughout the brain and body, with their various subtypes playing crucial roles in maintaining homeostasis and supporting cognitive development. Perioperative dyslipidemia can be regarded as a risk factor for neurological dysfunction. Changes in lipid homeostasis and metabolism can impact neuroinflammation, oxidative stress, and blood–brain barrier integrity, potentially contributing to the onset and progression of delirium (Shaw et al. 2025). Cardiac surgery may contribute to pathological changes such as intracerebral microembolism formation, cerebral hypoperfusion, decreased cerebral oxygen saturation, and inflammatory cytokine release, collectively forming the pathophysiological basis for the high incidence of POD following such procedures (Karu et al. 2010). Notably, a study identified 51 differentially expressed metabolites between elderly patients with and without POD after cardiopulmonary bypass (CPB) cardiac surgery, mainly enriched at the lipid and lipoprotein molecular levels (Huang et al. 2022). This finding indicates a strong link between POD following cardiac surgery and acute changes in lipid metabolism. Furthermore, preoperative administration of lipid-lowering drugs like simvastatin and fenofibrate has demonstrated potential protective effects against CPB-induced cognitive dysfunction and neuronal integrity impairment, with drugs are believed to exert their effects by protecting endothelial function and reducing inflammatory responses (Ouk et al. 2016).

AIP, as a simple indicator calculable through routine lipid testing (TG and HDL-C), sensitively assesses overall lipid profiles and lipoprotein patterns. It serves as a robust biomarker for predicting atherosclerotic cardiovascular disease, diabetes, and prediabetes by reflecting the balance between atherogenic and protective lipoproteins (Fernández-Macías et al. 2019; Fu et al. 2021; Zheng et al. 2022). Recent studies have further linked AIP to neurodegenerative disorders. Studies show a positive correlation between elevated AIP and the severity of white matter hyperintensities (WMH). WMH typically indicate microvascular ischemic changes in the brain, with clinical manifestations ranging from asymptomatic to cognitive impairment, dementia, or cerebrovascular accidents, including ischemic stroke and transient ischemic attacks (Kwon et al. 2022). Notably, conditions that increase cerebrovascular event risk (e.g., hypertension, atrial fibrillation, history of stroke) are themselves risk factors for POD (Guo et al. 2016; Oliveira et al. 2018). Although overt postoperative stroke is rare, imaging studies reveal that approximately 7–10% of elderly surgical patients exhibit evidence of asymptomatic cerebral ischemia, with these patients showing more than double the risk of POD (Mrkobrada et al. 2016; 2019). Concurrently, several cohort studies across various populations indicate a link between elevated AIP and the onset of cognitive impairment and dementia in middle-aged and elderly individuals (Huang et al. 2025; Lin et al. 2025), suggesting the need for further research into the relationship between AIP and perioperative cognitive outcomes.

The underlying regulatory mechanisms linking AIP and POD remain unclear, potentially involving multiple intertwined pathophysiological pathways. Elevated AIP not only reflects lipid metabolism disorders but is also closely associated with systemic inflammation and oxidative stress, both of which are core drivers of POD (Zhu et al. 2015). Elevated AIP typically indicates a relative increase in small dense low-density lipoproteins (sdLDL), these lipoproteins are more susceptible to oxidative modification and readily penetrate the vascular endothelium into the arterial wall, triggering macrophage phagocytosis and foam cell formation. This process initiates and amplifies inflammatory responses within the arterial wall (Mitsis et al. 2022). This lipid-driven state of low-grade chronic inflammation can be dramatically amplified under the stress of surgical trauma, leading to a cytokine storm that accelerates adverse effects on central nervous system function (Shaw et al. 2025). Conversely, HDL-C exerts multifaceted neuroprotective effects, including potent anti-inflammatory and antioxidant functions, as well as promoting the clearance of β-amyloid (Aβ) within the brain (Farhangrazi et al. 1997; Morris et al. 2021). Some studies suggest that higher HDL-C levels may suppress neuropathological inflammation and prevent cognitive impairment by modulating biochemical pathways through anti-inflammatory actions such as inhibiting antigen-presenting cell maturation and regulating lymphocyte inflammatory responses (Morris et al. 2021). Elevated AIP levels are frequently accompanied by reduced HDL-C, decreased cholesterol reverse transport, increased lipid accumulation, and enhanced proinflammatory effects (Yvan-Charvet et al. 2010). Consistent with existing studies, our results show that low HDL-C levels are associated with a higher risk of POD (Feinkohl et al. 2023). Oxidative stress represents another critical pathological mechanism in delirium development, characterized by reactive oxygen species (ROS) production and disruption of the brain's antioxidant defense system (Pang et al. 2022; Aguilar-Hernández et al. 2023). Higher oxidative stress may harms cellular structures such as lipids, proteins, and DNA, often resulting in neuronal impairment or cell death (Lopez et al. 2020). Elevated TG levels constitute another critical component of increased AIP. High TG levels impair brain health through multiple pathways, including inducing vascular endothelial dysfunction, exacerbating oxidative stress, and subsequently compromising BBB integrity. Increased BBB permeability facilitates the entry of peripheral inflammatory cells, cytokines, and harmful metabolic byproducts into the central nervous system (Shaw et al. 2025). Furthermore, hypertriglyceridemia may promote abnormal lipid deposition in the brain, impair cerebral blood flow autoregulation, and potentially accelerate Alzheimer's disease (AD)-related protein pathologies by affecting Aβ and tau metabolism (Lin et al. 2022; Burgess et al. 2006). A retrospective study revealed an association between elevated TG levels and increased delirium severity in elderly patients following hip fracture surgery (Chen et al. 2024). Therefore, AIP, as a composite indicator, may simultaneously capture dual risks: “weakened protective factors (e.g., HDL-C)” and “enhanced detrimental factors (e.g., atherogenicity).” This enables it to more accurately reflect the combined risks of neuroinflammation, oxidative stress, and cerebrovascular injury faced during surgical stress. This dual-risk capture may explain why AIP outperforms single lipid markers in predicting POD.

We performed subgroup analyses on various known or suspected risk factors for POD to further confirm the robustness of the association between AIP and POD. These included ICU length of stay, pre-existing comorbidities (e.g., cerebrovascular disease, neurological disease), anesthetic agents (e.g., dexmedetomidine, propofol), and surgery category. The findings indicated a consistent association between AIP and POD across all predefined subgroups, with no significant interactions detected. This indicates that the association between AIP and POD remains consistent across these diverse and important clinical factors. It further substantiates the potential of AIP as a generalizable biomarker applicable across patient populations with varying underlying disease states, different types of cardiac surgery, and diverse anesthesia management strategies.

Mediation analysis further suggests a potential mediating pathway linking AIP to POD. We found that RDW and Hb partially mediated the relationship between AIP and POD. Elevated AIP may promote inflammation and oxidative stress, impairing erythropoiesis and shortening red blood cell lifespan, leading to increased RDW and anemia (Zhou et al. 2015; Payne et al. 2004). This, in turn, causes insufficient oxygen supply to brain tissue. Inadequate oxygen transport may trigger alterations in cerebral blood flow, hypoperfusion, and impaired glucose metabolism, ultimately resulting in cerebrovascular lesions, accelerated amyloid deposition, and disruption of the blood–brain barrier, thereby increasing susceptibility to POD (Fornal et al. 2014). Furthermore, multiple population studies indicate that low Alb may be an independent risk factor for cognitive impairment and dementia (Hu et al. 2024). The negative correlation between AIP and serum Alb and its mediating role suggest that metabolic disorders represented by AIP may coexist with malnutrition/inflammatory states, collectively exacerbating patients' physiological vulnerability.

This research presents certain limitations. First, as a retrospective study, despite thorough comprehensive adjustments, the impact of unmeasured confounders like baseline cognitive function, social support levels, and surgical details(e.g.,duration of.

anesthesia, CPB time, transfusion volume). Second, data originated from a single center, necessitating prospective validation of conclusions across diverse populations and healthcare institutions. Third, ICD codes for delirium in MIMIC-IV are billing-oriented and may not fully correlate with bedside tools such as CAM-ICU, potentially underestimating true incidence. Fourth, we utilized only initial admission laboratory data, failing to dynamically observe the association between perioperative AIP changes and POD. Fifth, ICD codes in the MIMIC-IV database are recorded at the hospital-admission level and do not carry specific diagnostic timestamps; therefore, we were unable to determine whether the recorded diagnosis of delirium occurred during the ICU stay, the general ward period, or within a specific postoperative window. Finally, we are unable to establish causal relationships, whether lowering AIP or correcting dyslipidemia can effectively reduce the incidence of POD remains unproven and cannot be inferred from the present data. Future research should focus on validating AIP as a prognostic biomarker from prospective, multicenter cohorts researches, and on elucidating the underlying biological mechanisms linking lipid homeostasis to postoperative neurocognitive outcomes.

## Conclusion

In summary, this study provides that preoperative AIP is independently and nonlinearly associated with POD in patients undergoing cardiac surgery. As an easily calculable composite lipid indicator derived from routine lipid panels, AIP may offer a novel, readily accessible laboratory parameter for perioperative risk stratification, with the potential to aid in identifying patients at elevated risk of POD.

## Supplementary Information

Supplementary Material 1.

## Data Availability

Dataset of the study could be obtained from the MIMIC IV database (https://mimic. mit. edu).

## Abbreviations

AIP: Atherogenic Index of Plasma
POD: Postoperative Delirium
TG: Triglycerides
HDL-C: High-density Lipoprotein Cholesterol
MIMIC-IV: Medical Information from Critical Care
BIDMC: Beth Israel Deaconess Medical Center
NIH: National Institutes of Health
ICU: Intensive Care Unit
ICD-9/10: International Classification of Diseases, Ninth or Tenth Revision
SBP: Systolic Blood Pressure
DBP: Diastolic Blood Pressure
HR: Heart Rate
RR: Respiratory Rate
SpO_2_: Pulse Oximetry Oxygen Saturation
IQR: Interquartile Ranges
PSM: Propensity Score Matching
OR: Odds Ratios
CI: Confidence Intervals
RCS: Restricted Cubic Spline
SMDs: Standardized Mean Differences
Hb: Hemoglobin
Cl: Chloride
Alb: Albumin
RDW: Red Cell Distribution Width
WBC: White Blood Cell
BUN: Blood Urea Nitrogen
UA: Uric Acid
Cr: Creatinine
Glu: Glucose
BBB: Blood–Brain Barrier
CPB: Cardiopulmonary Bypass
WMH: White Matter Hyperintensities
sdLDL: Small Dense Low-density Lipoproteins
Aβ: β-Amyloid
ROS: Reactive Oxygen Species
AD: Alzheimer's Disease
