# Supplementary material for: The association between atherogenic index of plasma and postoperative delirium in cardiac surgery patients: an analysis of the MIMIC-IV database
Source: Perioper Med (Lond). 2026 Jul 8;15:85. doi: 10.1186/s13741-026-00717-x (PMC13632281; doi:10.1186/s13741-026-00717-x)
Supplement: Supplementary file 1 [file 13741_2026_717_MOESM1_ESM.docx]

**Table S1** ICD-9 and ICD-10 codes for identifying cardiacsurgeries and delirium.

|  | ICD-9 codes | ICD-10 codes |
| --- | --- | --- |
| Cardiac surgeries | 3511, 3512, 3514, 3521, 3522, 3523, 3524, 3527, 3611, 3612, 3613, 3614, 3615, 3616, 3619, 3845 | 0210089, 0210093, 0210099, 021009W, 02100A3, 02100A8, 02100A9, 02100AW, 02100Z3, 02100Z8, 02100Z9, 0211093, 0211099, 021109W, 02110A9, 02110AW, 02110Z3, 02110Z8, 02110Z9, 0212093, 0212099, 021209W, 02120AW, 02120Z8, 02120Z9, 0213093, 0213099, 021309W, 02130A3, 02130Z8, 02QF0ZZ, 02QG0ZZ, 02RF08Z, 02RF0JZ, 02RF0KZ, 02RG08Z, 02RG0JZ, 02RG0KZ, 02RJ08Z, 02RW0JZ, 02RX08Z, 02RX0JZ |
| Delirium | 29281, 2930, 2931, 2939, 34831, 34982, 78009, 78097 | F05, G92, G9341, R410, R4182 |

**Table S2** Analysis of the mediating role in delirium after cardiac surgery.

|  | **Total effect** | **Direct effect** | **Indirect effect** | **Mediation** |
| --- | --- | --- | --- | --- |
| Hb | 0.0593  (0.0472,0.0797) | 0.0613  (0.0453,0.0777) | -0.002(-0.00039,-0.005)0.04 | -3.37% |
| RDW | 0.0615  (0.0353,0.0846) | 0.06  (0.0343,0.0817) | 0.0015(0.0004,0.0031)0 | 2.09% |
| Lymphocyte | 0.0634  (0.0425,0.085) | 0.0643  (0.0437,0.0859) | -0.0009(-0.0022,0)0.08 | -1.29% |
| Monocyte | 0.0651  (0.0452,0.0871) | 0.0657  (0.0459,0.0874) | -0.0006(-0.0021,0.0013)0.56 | -1.1% |
| Neutrophil | 0.0634  (0.0359,0.0845) | 0.0625  (0.0346,0.0838) | 0.0009(-0.0012,0.0036)0.44 | 1.38% |
| WBC | 0.0636  (0.0384,0.088) | 0.0611  (0.0363,0.0849) | 0.0025(-0.0003,0.0048)0.12 | 4.16% |
| BUN | 0.0628  (0.0364,0.085) | 0.0618  (0.0362,0.0848) | 0.0009(-0.0001,0.0021)0.12 | 1.19% |
| UA | 0.0633  (0.0444,0.0828) | 0.0634  (0.0451,0.0828) | -0.0001(-0.0008,0.0007)0.8 | -0.04% |
| Cr | 0.0647  (0.0449,0.0889) | 0.064  (0.0446,0.0873) | 0.0007(-0.0004,0.0022)0.28 | 0.97% |
| Cl | 0.063  (0.0468,0.084) | 0.0644  (0.0477,0.087) | -0.0013(-0.0031,0.0001)0.12 | -1.82% |
| Alb | 0.0566  (0.0423,0.079) | 0.0601  (0.0388,0.0763) | -0.0035(-0.0064,-0.0011)0 | 6.18% |
| Glu | 0.0621  (0.0409,0.0831) | 0.0611  (0.0397,0.0819) | 0.0009(-0.0004,0.0021)0.12 | 1.4% |
| RR | 0.0649  (0.0465,0.0837) | 0.0634  (0.0455,0.0821) | 0.0015(0.0001,0.0035)0.04 | 2.11% |
| SpO_2_ | 0.063  (0.0385,0.0814) | 0.063  (0.0385,0.0813) | 0(0.0007,0.0007)0.84 | 0.03% |

Notes: Hb: Hemoglobin; RDW: Red Cell Distribution Width; WBC: White Blood Cell; BUN: Blood Urea Nitrogen; UA: Uric Acid; Cr: Creatinine; Cl: Chloride; Alb: Albumin; Glu: Glucose; RR: Respiratory Rate; SpO_2_: Pulse Oximetry Oxygen Saturation.
